# Autologous Stem Cell Therapy in Critical Limb Ischemia: A Meta-Analysis of Randomized Controlled Trials

**DOI:** 10.1155/2018/7528464

**Published:** 2018-05-24

**Authors:** Baocheng Xie, Houlong Luo, Yusheng Zhang, Qinghui Wang, Chenhui Zhou, Daohua Xu

**Affiliations:** ^1^Department of Pharmacology, Guangdong Medical University, Dongguan 523808, China; ^2^Institute of Laboratory Medicine, Guangdong Medical University, Dongguan 523808, China; ^3^School of Nursing, Guangdong Medical University, Dongguan 523808, China; ^4^Institute of Traditional Chinese Medicine and New Pharmacy Development, Guangdong Medical University, Dongguan 523808, China

## Abstract

**Objective:**

Critical limb ischemia (CLI) is the most dangerous stage of peripheral artery disease (PAD). Many basic researches and clinical treatment had been focused on stem cell transplantation for CLI. This systematic review was performed to review evidence for safety and efficacy of autologous stem cell therapy in CLI.

**Methods:**

A systematic literature search was performed in the SinoMed, PubMed, Embase, ClinicalTrials.gov, and Cochrane Controlled Trials Register databases from building database to January 2018.

**Results:**

Meta-analysis showed that cell therapy significantly increased the probability of ulcer healing (RR = 1.73, 95% CI = 1.45–2.06), angiogenesis (RR = 5.91, 95% CI = 2.49–14.02), and reduced the amputation rates (RR = 0.59, 95% CI = 0.46–0.76). Ankle-brachial index (ABI) (MD = 0.13, 95% CI = 0.11–0.15), TcO_2_ (MD = 12.22, 95% CI = 5.03–19.41), and pain-free walking distance (MD = 144.84, 95% CI = 53.03–236.66) were significantly better in the cell therapy group than in the control group (*P* < 0.01).

**Conclusions:**

The results of this meta-analysis indicate that autologous stem cell therapy is safe and effective in CLI. However, higher quality and larger RCTs are required for further investigation to support clinical application of stem cell transplantation.

## 1. Introduction

Critical limb ischemia (CLI) is the most dangerous stage of peripheral artery disease (PAD) caused by distal tissue hypoxia injury and lack of blood supply, including distal extremity ischemia, ulcers, or gangrene [1, 2]. The prevalence of PAD in the general population is 3% to 10% [3, 4]. The data showed that 11.2% of patients with PAD would deteriorate to CLI each year, and the patient with CLI had the high amputation and mortality rates [5]. Currently, patients in PAD could be treated by percutaneous transluminal angioplasty (PTA) or intravascular thrombolysis [6, 7]; however, 10%–30% of patients with CLI are not candidates for revascularization surgery. Many patients lose the chance of PTA, and the prognosis is poor after surgery, because the patients have peripheral atherosclerosis obliterans, extensive vascular disease, and/or serious damage caused by severe ischemic lesions of limbs [8, 9]. The studies [3, 10] found that vascular remodeling and other means still cannot alleviate the symptoms of ischemia. The amputation rate is 10%–40%, and the mortality rate is up to 20% in patients with CLI within 6 months [11]. The angiogenesis is the optimal treatment for CLI, and autologous stem cell therapy is an emerging alternative treatment [12, 13].

Since 2002, Tateishi-Yuyama et al. [14] have reported that bone marrow mononuclear cell transplantation was safe and effective for therapeutic angiogenesis in patients with CLI and it could significantly promote ulcer healing and reduce the amputation rate. During the past decades, a large number of basic researches and clinical treatment had been focused on stem cell transplantation for CLI [15]. The stem cell transplantation may improve pathophysiologic processes by stimulating the activities of tissue repair cells and inducing into vascular endothelial cells [16, 17]. However, only few evidences were available regarding safety and efficacy of autologous stem cell therapy in CLI. Meta-analyses have already become supporting evidence-based medicine. Although, there were some meta-analyses of stem cell therapy in CLI, the small amount of studies or incomplete indicators lead to the results of deviation and unconvinced [18, 19]. Therefore, this study of 23 RCTs with a total of 962 patients was included in order to acquire high-quality evidence for the clinical efficacy and safety of autologous stem cell therapy in CLI.

## 2. Methods

### 2.1. Literature Search

We searched the clinical studies, including SinoMed, PubMed, Embase, http://ClinicalTrials.gov, and Cochrane Controlled Trials Register databases from building database to January 2018. Using the terms number 1 “stem cells,” “mononuclear cells,” “granulocyte colony-stimulating factor,” “G-CSF,” “peripheral blood,” and “bone marrow,” the above search terms were connected with “OR”. Number 2 “critical limb ischemia,” “peripheral arterial disease,” “peripheral vascular disease,” “diabetic foot,” “revascularization,” “angiogenesis,” or “arteriogenesis”, the above search terms were connected with “OR”. Number 3 “randomized controlled”. Then, the above search terms of number 1, number 2, and number 3 were connected with “AND”. We manually searched the references of the original and review articles for possible related studies.

### 2.2. Study Selection

For the systematic review, we searched 23 clinical studies that met the following criteria: (1) patients with PAD or CLI, (2) received autologous stem cell therapy, (3) reported as randomized controlled trials (RCTs), (4) the control group received standard therapy with or without sham injections, (5) at least 1-month follow-up, and (6) reported efficacy and safety issues.

### 2.3. Data Extraction and Quality Assessment

Two of the authors independently extracted the data of literature and made a quality assessment process according to the predefined inclusion criteria. Difference among the two authors was solved by discussion with the third author. We used the Cochrane risk of bias tool for the quality evaluation of the included studies. This quality evaluating strategy included criteria concerning aspects of random sequence generation, allocation concealment, blinding of participants and personnel, blinding of outcome assessors, incomplete outcome data, selective reporting, and other biases [20].

### 2.4. Statistical Analysis

In this meta-analysis, statistical analysis was performed using RevMan software version 5.3 and we used risk ratio (RR) with 95% confidence interval (CI) for the analysis of dichotomous data, whereas the continuous data were presented as weighted mean difference (MD) or standardized mean difference (SWD) with 95% CI. Heterogeneity between the studies was determined using the chi-square test, with the *I*^2^ statistic, where *I*^2^ < 25% represent mild inconsistency, values between 25% and 50% represent moderate inconsistency and values > 50% suggest severe heterogeneity between the studies. We defined *I*^2^ > 50% as an indicator of significant heterogeneity among the trials. We used random effects' models to estimate the pooled results to minimize the influence of potential clinical heterogeneity among the studies, and the statistical significance was assumed at *P* < 0.05. Subgroup analysis was assessed using the *χ*^2^ test. Sensitivity analysis was performed to evaluate the robustness of merged results, by removing individual study. Publication bias was assessed by means of funnel plots.

## 3. Results

### 3.1. Search Results

**Figure 1:**
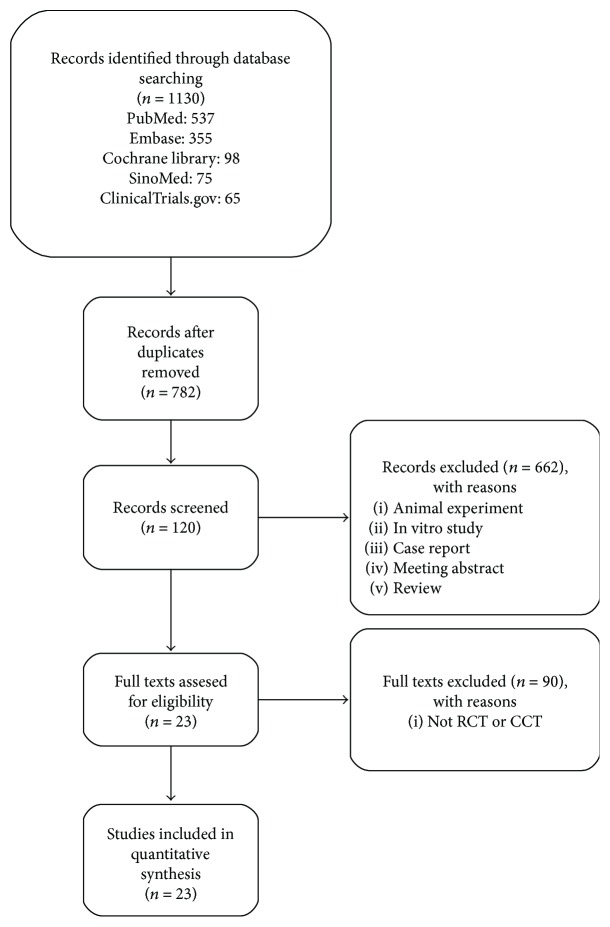
Flow chart and strategy of the meta-analysis.

A systematic search of studies published until January 2018 was performed through SinoMed, PubMed, Embase, http://ClinicalTrials.gov, and Cochrane Controlled Trials Register databases from building database. A total of 1130 literatures were searched, 23 RCTs were included in the inclusion criteria, and the literature search procedure was shown in Figure 1.

### 3.2. Study Characteristics

The general characteristics of the included studies were listed in Table 1. The included studies were 23 RCTs with a total of 962 patients. In these studies, the cell therapy group was one of the following stem cells: bone marrow mononuclear cells (BMMNCs, *n* = 7 studies), bone marrow mesenchymal stem cells (BMMSCs, *n* = 4 studies), bone marrow stem cells (BMSCs, *n* = 5 studies), peripheral blood mononuclear cells (PBMNCs, *n* = 2 studies), peripheral blood stem cells (PBSCs, *n* = 4 studies), CD34+ (*n* = 1 study), or CD133+ stem cells (*n* = 1 study). The transplantation method of stem cell was intramuscular (*n* = 20 studies) or intra-arterial (*n* = 3 studies). The patients in the control group received either placebo or standard care (*n* = 23 studies). The dose of stem cells was divided into three groups: high dose (10^9^, *n* = 5 studies), medium dose (10^8^, *n* = 5 studies), and low dose (10^7^, *n* = 5 studies). The mean follow-ups of the studies were 3 months (*n* = 9 studies), 6 months (*n* = 8 studies), and 12 months (*n* = 3 studies).

### 3.3. Quality Assessment

The risks of biases of the included studies were evaluated by the Cochrane assessment tool, and these results were summarized in Table 2. Three of the studies were at high risk of bias for blinding of participants and personnel and other biases according to the Cochrane Collaboration tool. Five studies reported methods of random sequence, and three studies reported the details of allocation concealment.

### 3.4. Amputation Rate

**Figure 2:**
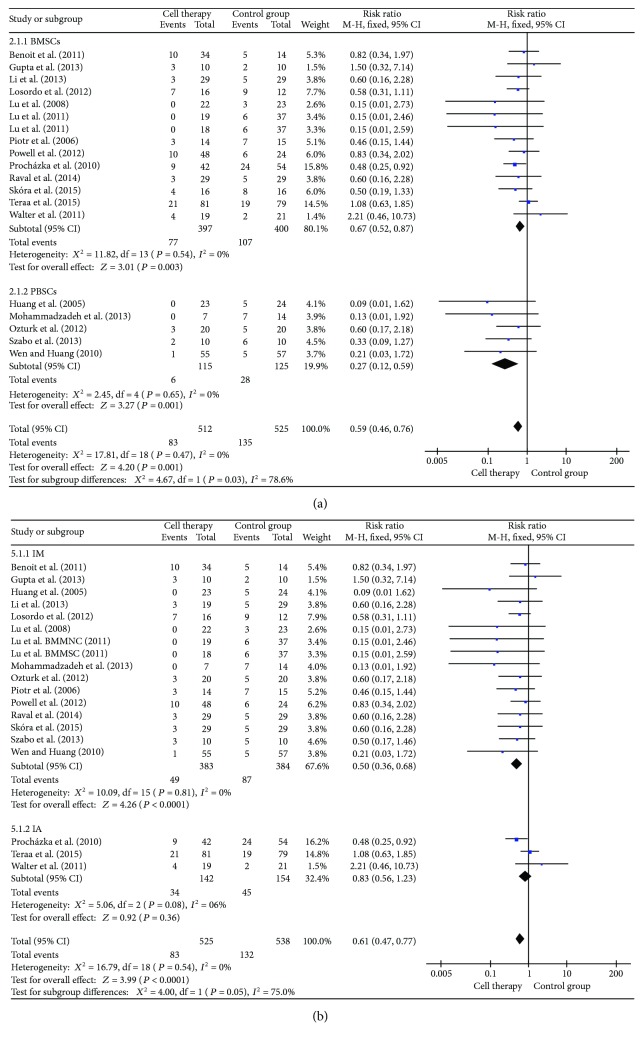
Forest plot of meta-analysis of the amputation rate in cell therapy and standard care for critical limb ischemia. (a) Subgroup analyses of bone marrow stem cells (BMSCs) versus peripheral blood stem cells (PBSCs). (b) Subgroup analyses of intramuscular (IM) versus intra-arterial (IA). Squares indicate the risk ratio, and horizontal lines represent 95% confidence intervals.

Amputation rate was reported in 18 studies with a total of 512 patients treated with cell therapy and 525 patients in the control groups (Figure 2). Cell therapy was associated with a significant 41% reduction in the amputation rate, compared with control groups (RR = 0.59, 95% CI = 0.46–0.76, *P* < 0.0001). Subgroup analyses indicated that peripheral blood stem cell (PBSC) was more beneficial than bone marrow stem cell (BMSC) on the amputation rate (*P* = 0.03, *I*^2^ = 78.6%). Intramuscular of autologous stem cell transplantation was better than intra-arterial in reducing the amputation rate (*P* = 0.05, *I*^2^ = 75%). The mean follow-ups of the studies were divided into 3 months, 6 months, and 12 months, and the group of 3 months was a significant difference compared with 6 months and 12 months (*P* = 0.03). Subgroup analysis among high dose (10^9^), medium dose (10^8^), and low dose (10^7^) showed that the group of low dose (10^7^) had a significant effect in reducing the amputation rate.

### 3.5. Ulcer Healing and Pain-Free Walking Distance

**Figure 3:**
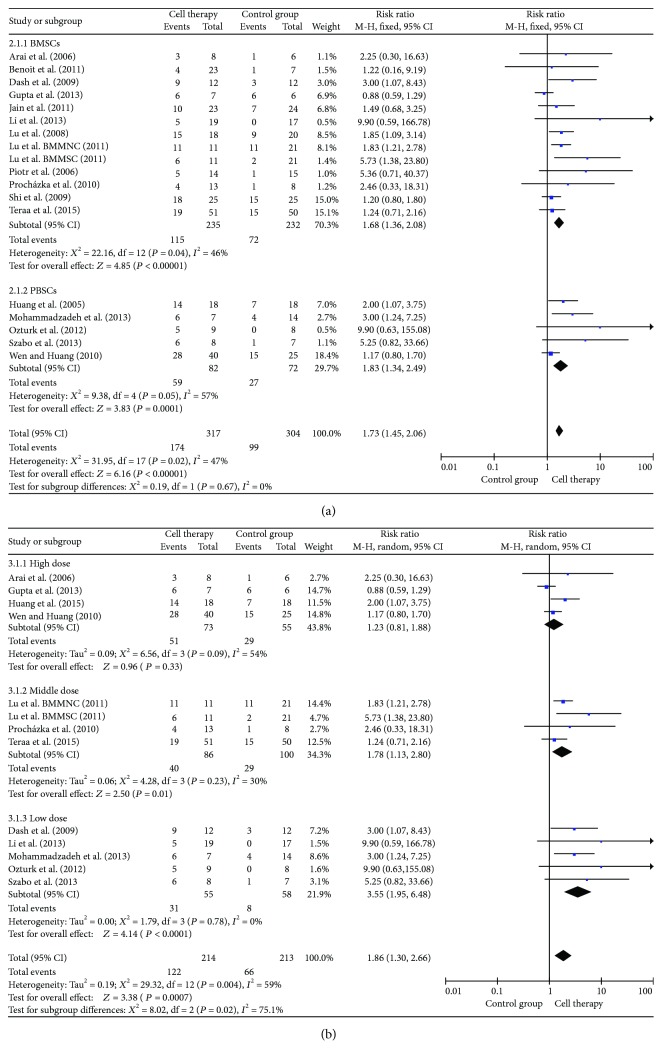
Forest plot of meta-analysis of ulcer healing in cell therapy and standard care for critical limb ischemia. (a) Subgroup analyses of bone marrow stem cells (BMSCs) versus peripheral blood stem cells (PBSCs). (b) Subgroup analyses among high dose (10^9^), medium dose (10^8^), and low dose (10^7^). Squares indicate the risk ratio, and horizontal lines represent 95% confidence intervals.

**Figure 4:**
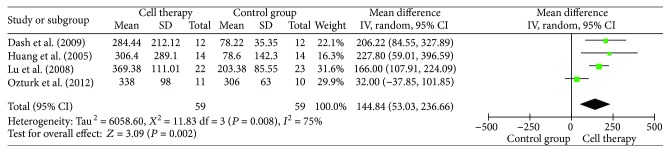
Forest plot of meta-analysis of pain-free walking distance in cell therapy and standard care for critical limb ischemia. Squares indicate the weighted mean difference, and horizontal lines represent 95% confidence intervals.

Ulcer healing was included in the analysis of 18 studies (Figure 3). Results of analysis showed that cell therapy could significantly increase the probability of ulcer healing (RR = 1.73, 95% CI = 1.45–2.06, *P* < 0.00001). Subgroup analyses indicated that the low dose (10^7^) group of autologous stem cell transplantation was better than the other groups in ulcer healing (RR = 3.55, 95% CI = 1.95–6.48, *P* = 0.02). Pain-free walking distance significantly increased in cell therapy (MD = 144.84, 95% CI = 53.03–236.66, *P* = 0.002) (Figure 4).

### 3.6. Ankle-Brachial Index (ABI) and Transcutaneous Oxygen Tension (TcO_2_)

**Figure 5:**
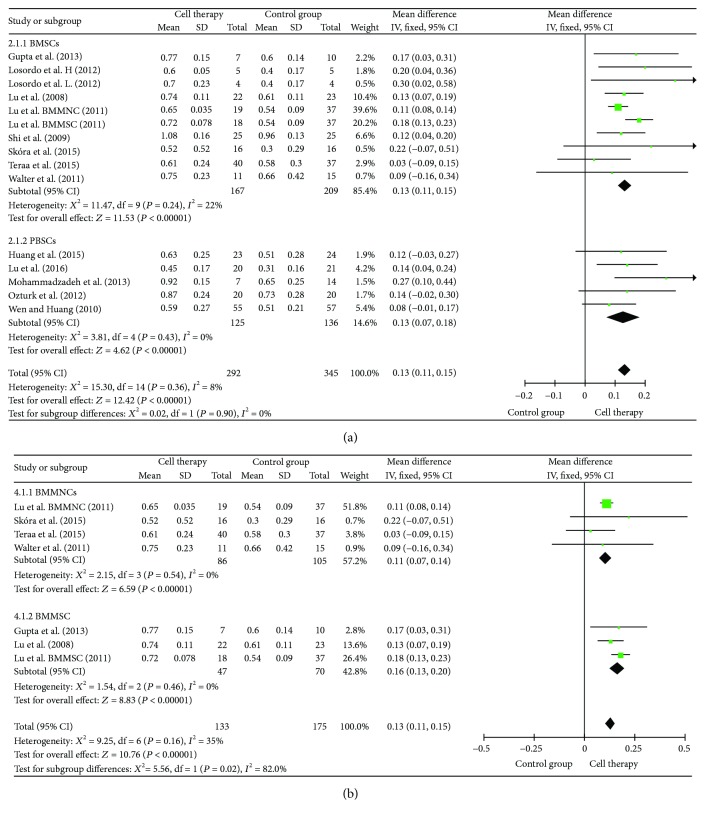
Forest plot of meta-analysis with the ankle-brachial index (ABI) in cell therapy and standard care for critical limb ischemia. (a) Subgroup analyses of bone marrow stem cells (BMSCs) versus peripheral blood stem cells (PBSCs). (b) Subgroup analyses among bone marrow mononuclear cells (BMMNCs) and bone marrow mesenchymal stem cells (BMMSCs). Squares indicate the weighted mean difference, and horizontal lines represent 95% confidence intervals.

**Figure 6:**
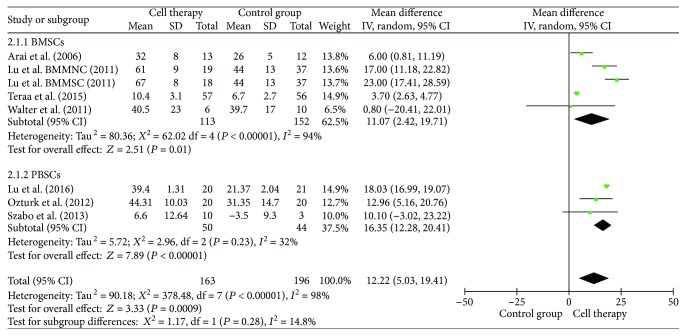
Forest plot of meta-analysis with transcutaneous oxygen tension (TcO_2_) in cell therapy and standard care for critical limb ischemia. Subgroup analyses of bone marrow stem cells (BMSCs) versus peripheral blood stem cells (PBSCs). Squares indicate the weighted mean difference, and horizontal lines represent 95% confidence intervals.

ABI with 15 studies was included in the analysis (Figure 5). Results indicated that cell therapy significantly improved the ABI by 0.13 (MD = 0.13, 95% CI = 0.11–0.15, *P* < 0.00001). Subgroup analyses indicated that bone marrow mesenchymal stem cells (BMMSCs) were superior to bone marrow mononuclear cells (BMMNCs), but there was no significant difference between bone marrow stem cells (BMSCs) and peripheral blood stem cells (PBSCs) in improving the ABI. The TcO_2_ with 8 studies was included in the analysis. Results indicated that cell therapy significantly improved TcO_2_ by 12.22 mmHg (MD = 12.22, 95% CI = 5.03–19.41, *P* = 0.0009). Subgroup analyses showed that there was no beneficial effect between BMSCs and PBSCs on the TcO_2_ (Figure 6).

### 3.7. Angiogenesis and Blood Flow of 10 Toes

**Figure 7:**
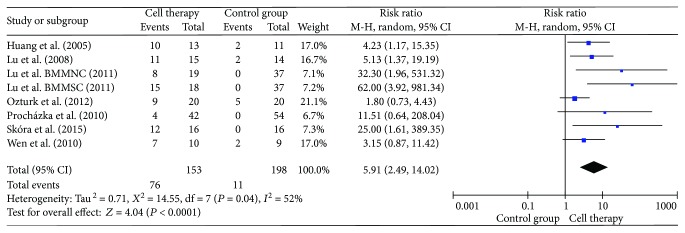
Forest plot of meta-analysis with angiogenesis in cell therapy and standard care for critical limb ischemia. Squares indicate the risk ratio, and horizontal lines represent 95% confidence intervals.

**Figure 8:**
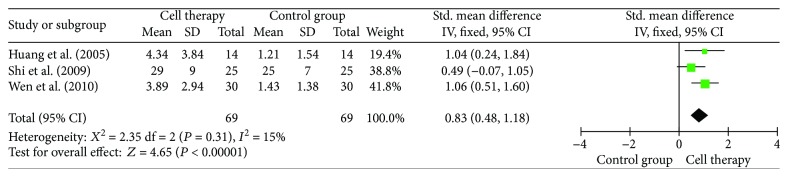
Forest plot of meta-analysis with blood flow of 10 toes in cell therapy and standard care for critical limb ischemia. Squares indicate the standardized mean difference, and horizontal lines represent 95% confidence intervals.

There were 8 studies included in the analysis with angiogenesis (Figure 7). Analysis by digital subtraction angiography revealed that autologous stem cell transplantation significantly improved the new vessel form (RR = 5.91, 95% CI = 2.49–14.02, *P* < 0.0001). The number of ischemic limbs with rich new collateral vessels in the transplant patients was significantly higher than that in the control group. Meanwhile, the blood flow of 10 toes significantly increased in cell therapy (SMD = 0.83, 95% CI = 0.48–1.18, *P* < 0.00001) (Figure 8).

### 3.8. Publication Bias and Heterogeneity

**Figure 9:**
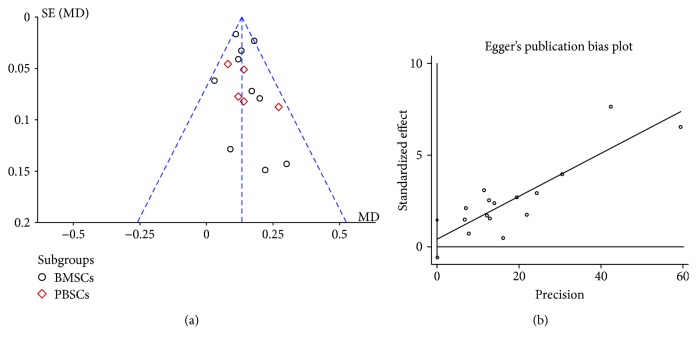
Meta-analysis of publication bias of the ankle-brachial index (ABI) in cell therapy and standard care for critical limb ischemia. (a) Funnel plot of the ABI. (b) Egger's funnel plot of the ABI.

**Figure 10:**
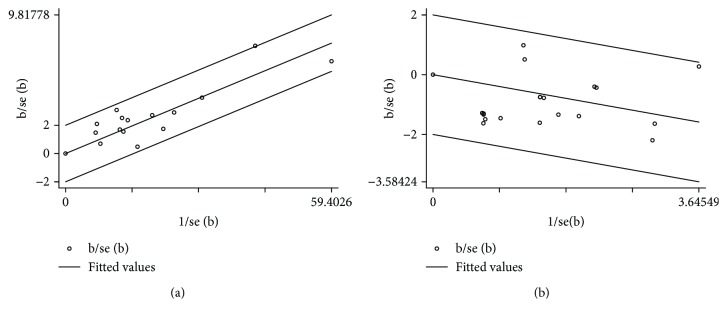
Meta-analysis of sensitivity in cell therapy and standard care for critical limb ischemia. (a) Galbraith plot of the ankle-brachial index (ABI). (b) Galbraith plot of the amputation rate.

According to this meta-analysis, the significant symmetry showed that the ABI did not have obvious publication bias. Furthermore, the Egger's test funnel plot also indicated that there was no obvious publication bias in the ABI (*P* > 0.363, 95% CI = −0.57–1.45) (Figure 9). Sensitivity analysis was performed using a Galbraith plot for all the indicators. The results showed that there was no substantial change in the ABI and amputation rate, indicating that the results of meta-analysis were credible (Figure 10).

## 4. Discussion

### 4.1. Main Outcome

The registrations of stem cell clinical trials were retrieved on USA National Institutes of Health (NIH) clinical trial registration website (http://ClinicalTrials.gov). We performed the databases from building database to January 2018. There were 4715 clinical trial registration information for stem cells all over the world, and there were 2399 studies in America, 1027 studies in Europe, and 574 studies in China. We analyzed the disease of stem cell therapy and found that there were 1767 studies on neoplasms by histologic type, 1279 studies on immune system diseases, 607 studies on vascular diseases, and 513 studies on bone marrow diseases. The data showed that stem cell therapy has been used in various diseases, and stem cell therapy is approved in the globe. This meta-analysis included 23 RCTs with a total of 962 patients with CLI who were ineligible for surgical or percutaneous revascularization. Results indicated that autologous stem cell therapy had the potential effect to reduce the risk of amputation by 41% and significantly increased the probability effect of ulcer healing by 73% compared with the control group. ABI and TcO_2_ are the basic indicators of CLI, and the results indicated that cell therapy significantly improved the ABI by 0.13 and TcO_2_ by 12.22 mmHg. Moreover, the value of the increased ABI and TcO_2_ level were meaningful to confirm the truth of the improvements of amputation and wound healing rates. In addition, cell therapy could improve the endpoints of limb perfusion, and the blood flow of 10 toes significantly increased in cell therapy, compared with the control group. We speculated that the main reason for the increases of limb perfusion was angiogenesis. The studies reported that endothelial progenitor cells (EPCs) derived from the bone marrow can facilitate microvasculature regeneration by paracrine or direct mechanisms in regions of blood vessel formation [21, 22]. Therefore, we made a statistics on the use of angiography in patients with CLI. There were 8 studies with RCTs in the analysis, revealing a significant effect of angiogenesis after autologous stem cell transplantation.

### 4.2. Subgroup Analysis

A study by Tateishi-Yuyama et al. [14] reported that transplantation of bone marrow stem cell therapy in patients with CLI significantly improved TcO_2_, ABI, and pain-free walking distance. Hereafter, many studies with RCTs had investigated the safety and feasibility of autologous stem cells of BMMNC therapy in CLI [5, 15, 23–26]. In recent years, a variety of cell types have been studied for treatment of PAD or CLI, including PBSCs, BMSCs, BMMNCs, PBMNCs, and BMMSCs. Our subgroup analyses indicated that PBSCs were more beneficial than BMSCs on the amputation rates. Dubsky et al. [13, 27] suggested that there was no significant difference in long-term prognosis between patients treated with BMMNCs and those treated with PBMNCs. The trials reported that transplantation of BMMSCs was safe and no serious adverse events by cell injection after the follow-up period [28, 29]. RCTs by Lu et al. [15] suggested that ulcer healing, ABI, TcO_2_, painless walking time, and magnetic resonance angiography (MRA) in the BMMSC group were significantly higher than that in the BMMNC group in diabetic patients with CLI. The subgroup analyses indicated that BMMSCs showed beneficial effect than BMMNCs in improving the ABI. Therefore, BMMSCs could be more effective than BMMNCs in the treatment of CLI.

In RCTs of patients with CLI, the most common route of stem cell therapy administration was intramuscular. But, the potential route of intra-arterial was also injected therapy [5, 25, 30]. In order to find suitable and beneficial injection therapy, we conducted subgroup analysis. The results showed that the amputation rate in the intramuscular group was significantly lower than that in the intra-arterial group. The JUVENTAS trial is the largest RCT to investigate the effects of BMMNCs by intra-arterial [5]. The study [5] reported that repetitive intra-arterial of autologous BMMNCs was not effective in reducing the primary outcome of the amputation rate at 6 months, ABI, ulcer healing, and TcO_2_. Therefore, we suggest that stem cell administration is suitable and beneficial choice by intramuscular injection. In addition, we found that the low dose (10^7^) group was a significant difference on the amputation rate compared with high dose (10^9^) and medium dose (10^8^) groups (*P* = 0.03), and cell therapy with low dose (10^7^) significantly reduced the amputation rate. The cell therapy with low dose (10^7^) showed a significant improvement in ulcer healing in patients with CLI [26, 31, 32]. However, a degree of heterogeneity may be generated in subgroup analysis, which could negatively impinge upon the assessment on efficacy of cell therapy. The generated heterogeneity could mask the true effect of cell therapy [10]. So we think that the results of subgroup analysis need the large clinical trials as evidence to support.

### 4.3. Safety

The studies of 23 RCTs showed that cell therapy was relatively safe, and the adverse events were mostly mild and transient. Teraa et al. [5] reported that there was a patient with inguinal hematoma due to intra-arterial injection, and the study of Szabo et al. [33] found that the cell therapy group had three adverse events during 3 months, but there was no evidence that the adverse events were attributed to stem cell transplantation. Li et al. [26] reported that there are three patients with fever in the cell therapy group, and they were cured after treatment. Lu et al. [15] showed that a few patients had a short-term response of mild pain 2 hours after cell transplantation, but no complications were detected, such as immune rejection and allergic reactions. Wen and Huang [34] reported that some patients felt uncomfortable of their limbs after intramuscular injection of PBSCs within 1 week, and the intramuscular injection site did not appear infected during 3-month follow-up. Similarly, many studies reported that stem cell transplantation was safe in long-term follow-up [28, 35]. The study by Molavi et al. [36] showed no adverse events during the 24-week follow-up period after cell delivery. No serious adverse events were found in the 23 studies included in this meta-analysis. Therefore, autologous stem cell transplantation is safe in the treatment of CLI.

In conclusion, this meta-analysis suggests that autologous stem cell therapy is safe and effective in CLI. Subgroup analysis indicates that cell types, cell dosage, route of administration, and follow-up time are the very important factors in stem cell therapy. However, we still lack high quality and large scale of RCTs to explore the influence of factors and the effect of autologous stem cell therapy in CLI.

## Figures and Tables

**Table 1:** Characteristics of included clinical studies.

Study	Sample (T/C)	Age (T/C)	Intervention		Injection	Follow-up	Number of cells	Evaluation
			T	C				
Huang et al. [37]	14/14	71.1/70.9	PBMNCs	Standard care	IM	12 w	3 × 10^9^	①, ②, ③, ④, ⑥, ⑦
Arai et al. [23]	13/12	62/68	BMMNCs	Standard care	IM	1 mo	1–3 × 10^9^	②, ⑤
Barć et al. [24]	14/15	Unclear	BMMNCs	Standard care	IM	6 mo	Unclear	①, ②
Lu et al. [38]	22/23	66.6/65.5	BMMSCs	Standard care	IM	12 w	7.32 × 10^8^–5.61 × 10^9^	①, ②, ③, ④, ⑥
Dash et al. [39]	12/12	40	BMMSCs	Standard care	IM	12 w	4.5-6 × 10^7^	②, ⑥
Shi et al. [40]	25/25	Unclear	BMSCs	Standard care	IM	3 mo	Unclear	②, ④, ⑦
Procházka et al. [30]	42/54	66.2/64.1	BMSCs	Standard care	IA	4 mo	1.96 × 10^8^	①, ②, ③
Wen and Huang [34]	30/30	63	PBSCs	Standard care	IM	3 mo	3 × 10^9^	①, ②, ③, ④, ⑦
Lu [15]	21/41	63	BMMNCs	Standard care	IM	24 w	9.3 × 10^8^	①, ②, ③, ④, ⑤
Lu et al. [15]	20/41	65	BMMSCs	Standard care	IM	24 w	9.6 × 10^8^	①, ②, ③, ④, ⑤
Walter et al. [25]	19/21	64.4/64.5	BMMNCs	Standard care	IA	6 mo	1.53 × 10^8^	①, ④, ⑤
Jain et al. [41]	25/23	54/58	BMSCs	Standard care	IM	3 mo	Unclear	②
Benoit et al. [42]	34/14	65.7/72.5	BMSCs	Standard care	IM	6 mo	Unclear	①, ②
Losordo et al. [43]	16/12	66.2/67.1	CD34+	Standard care	IM	12 mo	1 × 10^6^ 1 × 10^5^	①, ④
Powell et al. [44]	48/24	67.3/69.2	BMSCs	Standard care	IM	12 mo	0.35–2.95 × 10^8^	①
Ozturk et al. [31]	20/20	71.9/70.8	PBMNCs	Standard care	IM	3 mo	2.48 × 10^7^	①, ②, ③, ④, ⑤, ⑥
Gupta et al. [29]	10/10	43/47.6	BMMSCs	Standard care	IM	6 mo	2 × 10^9^	①, ②, ④
Li et al. [26]	29/29	61/63	BMMNCs	Standard care	IM	6 mo	1 × 10^7^	①, ②
Mohammadzadeh et al. [32]	7/14	63.5/64.2	PBSCs	Standard care	IM	3 mo	2 × 10^7^	①, ②, ④
Szabo et al. [33]	10/10	60.6/63	PBSCs	Standard care	IM	24 mo	6.64 × 10^7^	②, ⑤
Raval et al. [9]	7/3	65/85	CD133+	Standard care	IM	12 mo	5–40 × 10^7^	①
Teraa et al. [5]	81/79	69/65	BMMNCs	Standard care	IA	6 mo	5-6 × 10^8^	①, ②, ④, ⑤
Skóra et al. [45]	16/16	66.7/68.3	BMMNCs	Pentoxifylline	IM	3 mo	1.58 × 10^9^	①, ③, ④
Lu et al. [46]	20/21	67.2	PBSCs	Standard care	IM	6 mo	Unclear	④, ⑤

Note: T = cell therapy; C = control group; IM = intramuscular; IA = intra-arterial; w = week; mo = month; PBMNCs = peripheral blood mononuclear cells; BMMNCs = bone marrow mononuclear cells; BMMSCs = bone marrow mesenchymal stem cells; BMSCs = bone marrow stem cells; PBSCs = peripheral blood stem cells; ① = amputation; ② = ulcer healing; ③ = angiographic; ④ = ABI; ⑤ = TcO_2_; ⑥ = pain-free walking distance; ⑦ = the blood flow of 10 toes.

**Table 2:** Cochrane risk of bias assessment.

Study	Random sequence generation	Allocation concealment	Blinding of participants and personnel	Blinding of outcome assessment	Incomplete outcome data	Selective reporting	Other biases
Huang et al. [37]	Unclear	Unclear	Unclear	Unclear	Low	Unclear	Low
Arai et al. [23]	Unclear	Unclear	High	Unclear	Low	Low	Low
Barć et al. [24]	Unclear	Unclear	High	Unclear	Low	Low	Low
Lu et al. [38]	Unclear	Unclear	Unclear	Unclear	Low	Low	Low
Dash et al. [39]	Unclear	Unclear	High	Unclear	Low	Unclear	Low
Shi et al. [40]	Unclear	Unclear	High	Unclear	Low	Low	High
Procházka et al. [30]	Low	Low	High	Unclear	Low	Low	Low
Wen and Huang [34]	Unclear	Unclear	Unclear	Unclear	Low	Low	High
Lu [15]	Unclear	Unclear	Low	Unclear	Low	Unclear	Low
Lu et al. [15]	Low	Unclear	Low	Low	Low	Low	Low
Walter et al. [25]	Unclear	Unclear	Low	Unclear	Low	Unclear	Low
Jain et al. [41]	Low	Low	Low	Unclear	Low	Unclear	Low
Benoit et al. [42]	Unclear	Unclear	Low	Unclear	Low	Low	Low
Losordo et al. [43]	Unclear	Unclear	Low	Low	Low	Unclear	Low
Powell et al. [44]	Unclear	Unclear	Low	Unclear	Low	Unclear	Low
Ozturk et al. [31]	Unclear	Unclear	High	Unclear	Low	Low	Low
Gupta et al. [29]	Low	Low	Low	Low	Low	Low	Low
Li et al. [26]	Unclear	Unclear	Unclear	Unclear	Low	Unclear	Low
Mohammadzadeh et al. [32]	Unclear	Unclear	Unclear	Unclear	Low	Low	Low
Szabo et al. [33]	Unclear	Unclear	High	Low	Low	Low	Low
Raval et al. [9]	Unclear	Unclear	Low	Low	Low	Low	Low
Teraa et al. [5]	Low	Unclear	Low	Low	Low	Low	Low
Skóra et al. [45]	Low	Unclear	Low	Low	Low	Low	Low
Lu et al. [46]	Unclear	Unclear	High	Low	Low	Low	High

Note: low = low risk of bias; unclear = unclear risk of bias; high = high risk of bias.
